# Anti-tumor activity of an immunotoxin (TGFα-PE38) delivered by attenuated *Salmonella typhimurium*

**DOI:** 10.18632/oncotarget.17197

**Published:** 2017-04-18

**Authors:** Daejin Lim, Kwang Soo Kim, Hyunju Kim, Kyong-Cheol Ko, Jae Jun Song, Jong Hyun Choi, Minsang Shin, Jung-joon Min, Jae-Ho Jeong, Hyon E. Choy

**Affiliations:** ^1^ Department of Microbiology, Chonnam National University Medical School, Gwangju, Republic of Korea; ^2^ Molecular Medicine, BK21 Plus, Chonnam National University Graduate School, Gwangju, Republic of Korea; ^3^ Department of Nuclear Medicine, Chonnam National University Medical School, Gwangju, Republic of Korea; ^4^ Applied Microbiology Research Center, Bio-Materials Research Institute, Korea Research Institute of Bioscience and Biotechnology, Jeongeup, Jeonbuk, Republic of Korea; ^5^ Department of Microbiology, Kyungpook National University Medical School, Daegu, Republic of Korea

**Keywords:** immunotoxin, TGFα-PE38, bacterial cancer therapy, Salmonella

## Abstract

The anticancer strategy underlying the use of immunotoxins is as follows: the cancer-binding domain delivers the toxin to a cancer cell, after which the toxin enters and kills the cell. TGFα-PE38 is an immunotoxin comprising transforming growth factor alpha (TGFα), a natural ligand of epidermal growth factor receptor (EGFR), and a modified *Pseudomonas* exotoxin A (PE38) lacking N terminal cell-binding domain, a highly potent cytotoxic protein moiety. Tumor cells with high level of EGFR undergo apoptosis upon treatment with TGFα-PE38. However, clinical trials demonstrated that this immunotoxin delivered by an intracerebral infusion technique has only a limited inhibitory effect on intracranial tumors mainly due to inconsistent drug delivery. To circumvent this problem, we turned to tumor-seeking bacterial system. Here, we engineered *Salmonella typhimurium* to selectively express and release TGFα-PE38. Engineered bacteria were administered to mice implanted with mouse colon or breast tumor cells expressing high level of EGFR. We observed that controlled expression and release of TGFα-PE38 from intra-tumoral *Salmonellae* by either an engineered phage lysis system or by a bacterial membrane transport signal led to significant inhibition of solid tumor growth. These results demonstrated that delivery by tumor-seeking bacteria would greatly augment efficacy of immunotoxin in cancer therapeutics.

## INTRODUCTION

Several recombinant immunotoxins developed to target malignant tumors are now undergoing clinical trials [1, 2]. Immunotoxins comprise a cancer-binding moiety linked to a potent toxin lacking an intrinsic cell-binding domain. The cancer-binding moiety most often comprises part of a monoclonal antibody, although cytokines or growth factors that interact with receptors highly expressed by cancer cells have also been used. The idea behind anticancer strategies based on immunotoxins is that the cancer-binding moiety brings the toxin to cancer cells; the toxin then enters and kills the cells. Many tumor cells express high levels of epidermal growth factor receptor (EGFR) [3]. Transforming growth factor alpha (TGFα) is a natural ligand for the EGFR, which plays a central role in cancer development. A recombinant immunotoxin comprising TGFα and a modified *Pseudomonas* exotoxin A (PE38) derived from *Pseudomonas aeruginosa* was developed for treatment of EGFR-expressing malignant tumors, *e.g*. brain tumors [4, 5]*. Pseudomonas* exotoxin A acts by inactivating protein synthesis in mammalian cells [6]. PE38, which lacks an intrinsic cell-binding domain, binds to EGFR-expressing cancer cells via the TGFα moiety within the recombinant toxin. It has been demonstrated that the TGFα-PE38 fusion protein was cytotoxic to EGFR-expressing tumor cells *in vitro* and in xenograft mouse models [1, 7]. However, there are some limitations. For example, dose-limiting hepatotoxicity was noted when high levels of TGFα-PE38 were administered systemically [7]. It was suggested that a direct intra-tumoral drug delivery could ensure successful application of TGFα-PE38 for the treatment of solid tumors, including intracranial glioblastoma. Thus, TGFα-PE38 was delivered directly to the tumor to treat intracranial implants of glioblastoma cells in nude mice. Intra-tumoral delivery was imperative to avoid the hepatotoxicity but also to solve a problem of its short half-live (the analogous construct TGFα-PE40 has a half-life of 10–20 min [8]). In a subsequent clinical trial of human patients with recurrent malignant brain tumors, TGFα-PE38 was delivered by an intracerebral infusion technique [9]. However, a limited positive response was observed, mainly due to inconsistent drug delivery by this technique.

Bacterial strains from several phylogenetic groups, including *Salmonella*, *Clostridium*, *Bifidobacterium*, *Listeria*, *Vibrio*, and *Escherichia coli (E. coli)*, selectively target and proliferate within solid tumors in mouse models [10–15]; indeed, bacterial anticancer therapy was developed to take advantage of these properties. Recently, bacterial anticancer therapy using a mutant *Salmonella typhimurium* was clinically tested in canines and in human patients [16–18]. As cancer therapeutic agents, bacteria possess several advantages [19–22]. First, these bacteria preferentially overgrow within tumors, resulting in ~1,000-fold (or even higher) increase in bacterial numbers in tumor tissues relative to normal organs such as the liver and spleen. Second, they can actively swim away from the vasculature and penetrate deep into tumor tissue, keeping high concentration in hypoxic tumor tissue. Third, native bacterial cytotoxicity can suppress tumor growth. Fourth, the oncolytic effect of bacteria is significantly enhanced if tumor-targeting bacteria are armed with cytolytic proteins such as bacterial cytolysin (CytA). Recently, R. Hoffman's group, which has been used a modified auxotrophic strain of *Salmonella typhimurium* A1R rather successfully by itself to eradicate metastatic as well as primary tumors [14, 23–30], also began combinatorial approach with chemotherapy after finding that A1R strain decoyed chemo-resistant quiescent cancer cells in tumors to cycle from G0/G1 to S/G2/M, thereby rendering these cells sensitive to cytotoxic agents: a new paradigm of “decoy, trap and shoot” chemotherapy [28–30].

Here, we have used ΔppGpp mutant *Salmonellae* armed with recombinant TGFα-PE38 to treat solid tumors. This strain of bacteria is incapable of invading or proliferating in animal cell [31, 32] but it alone has been shown to be anti-tumoral, although temporal, by inducing expression of pro-inflammatory cytokines, interleukin-1β and tumor necrosis factor α, expressed by intra-tumoral macrophages and neutrophils [33]. In this study, we constructed a plasmid harboring TGFα-PE38, which was then expressed in this *Salmonellae* targeted to tumors implanted in mice using an induction system based on the *P*_BAD_ promoter from *E. coli*; this promoter is inducible by intraperitoneal (i.p.) injection of L-arabinose [20]. TGFα-PE38 was exported out of *Salmonellae* either by an engineered phage lysis system [34] or by a bacterial membrane transport signal fused to the protein. The results of the animal studies showed that controlled expression and release of TGFα-PE38 from *Salmonella* resulted in significant retardation of tumor growth better than the *Salmonella* alone.

## RESULTS

### Construction and analysis of plasmids expressing TGFα-PE38, SEC–TGFα-PE38, and PE38

**Figure 1:**
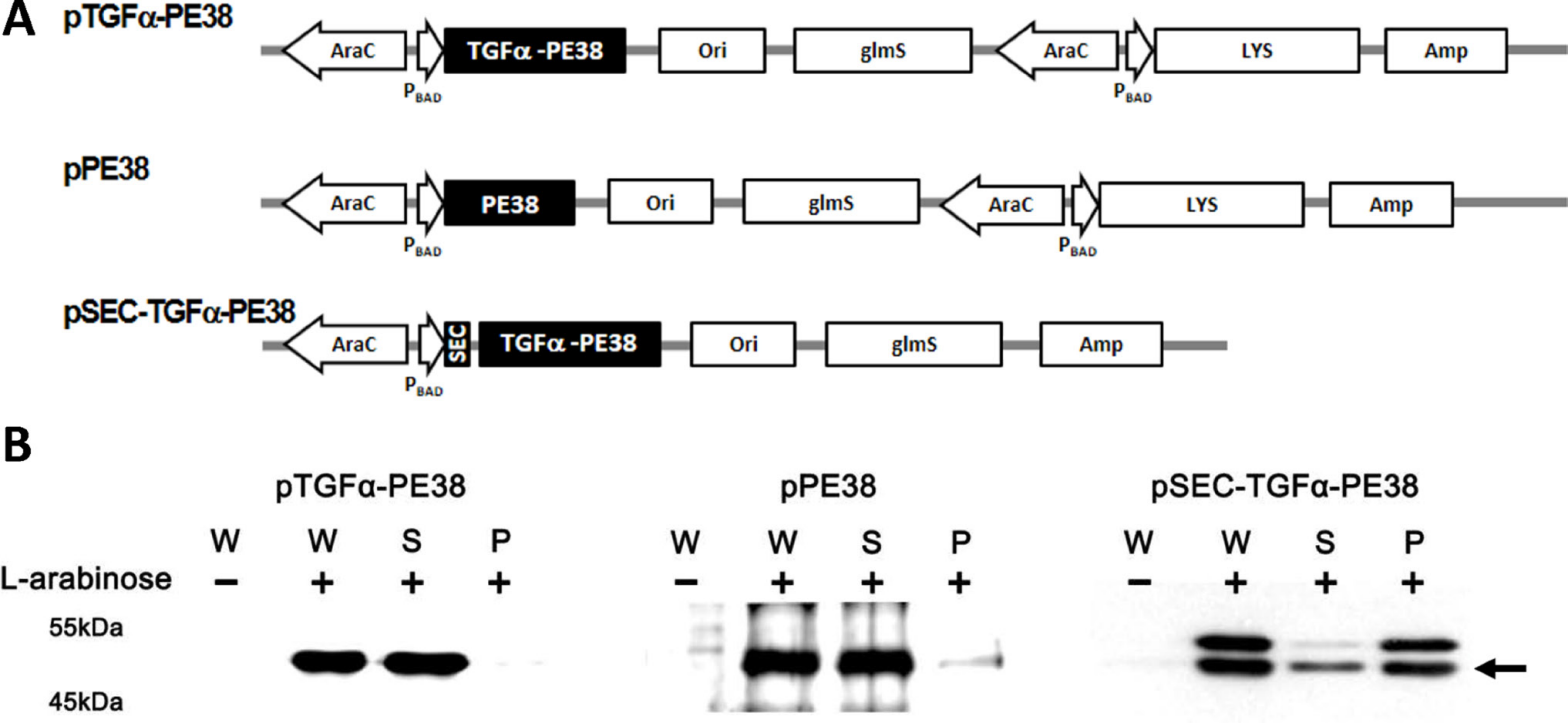
(**A**) Map of the plasmids used in this study: *pTGFα-PE38* carries the 8293bp *TGFα*-PE38 DNA fragment under control of the P_BAD_ promoter on a *pLYS* background [34]; *pPE38* is the same as above, but lacks *TGF*α DNA; *pSEC-TGFa-PE38* is the same as *pTGFα-PE38* but carries a secretion sequence before the *TGF*α DNA and lacks LYS (a phage lysis gene). (**B**) ΔppGpp *S. typhimurium* carrying one of these plasmids was grown overnight in LB medium, diluted 50-fold in the same medium, and grown for 2 hours. L-arabinose was added at a final concentration of 0.2%. Samples were taken after 1 hour, and expression and release of cargo protein from the *Salmonellae* carrying the above plasmids were examined. The bacterial culture (W) was split into supernatant (S) and pellet (P) fractions by centrifugation (3,000 × g/15 min). TGFα-PE38/PE38 was examined by Western blot analysis using antibody specific for *Pseudomonas* exotoxin A. Arrow indicates mature product.

In bacterial anticancer therapy, it is essential to maintain the plasmid carrying the gene encoding the oncolytic protein in the absence of selection pressure (e.g., via antibiotics) in animals. The plasmid should therefore be equipped with a balanced-lethal host vector system [35]. The current study used a system based on the *glmS* gene, which is essential for peptidoglycan synthesis in *Salmonella* [36]. Mutants defective in *glmS* are strictly dependent on the presence of exogenous D-glucosamine (GlcN) and N-acetyl-D-glucosamine (GlcNAc). Since these compounds are not present in mammalian tissues, this balanced-lethal system requires that *Salmonellae* carry the recombinant *GlmS*^+^ plasmid to survive. In addition, recombinant oncolytic proteins expressed in bacteria must be exported out of bacteria to be effective against tumors. Previously, we reported that induction of a plasmid (*pLYS*) carrying a *Salmonellae* lysis system consisting of three genes from a *Salmonella* bacteriophage (iEPS5) effectively lysed bacteria and released their contents [34]. On this plasmid background (*pLYS*), a DNA fragment containing the open reading frame of TGFα-PE38 or PE38 was cloned under control of the inducible *araBAD* promoter of *E. coli* to generate *pTGFα-PE38* or *pPE38* (Figure 1A). Alternatively, we intended to take an advantage of bacterial signal peptide to export TGFα-PE38 out of *Salmonella* cells [37]. To identify the optimal signal peptide for export of TGFα-PE38, we tested various signal peptides, including the pelB leader sequence of the gene for pectatelyase B from *Erwiniacarotovora* [38], CelEx-BR12 [39], and a new cellulase (Psp) from *Paenibacillus* sp. EC003, which was isolated from soil as a cellulolytic microorganism that formed a clear zone on LB agar plates containing Azo-CM-cellulose (manuscript in preparation). The Psp signal peptide comprised of 32 amino acids (MGLKMKKRSGKKAWMLLVMSLLIAAVPITASA) allowed efficient export of TGFα-PE38 (see below). A plasmid carrying the Psp secretion signal, fused in-frame to the N´ end of TGFα-PE38 was constructed in the same way, on the *pLYS* plasmid background lacking the phage lysis system; this plasmid was named pSEC-TGFα-PE38. TGFα-PE38 fused to this signal peptide should be secreted out of bacteria upon induction by L-arabinose. ΔppGpp *S. typhimurium* was used, since it is highly attenuated in mouse models [32, 40].

Using *Salmonellae* carrying the above plasmid constructs, we then examined the controlled induction of TGFα-PE38 and/or bacterial lysis *in vitro*. ΔppGpp *Salmonella* carrying these constructs were grown in LB medium. Two hours after inoculation, when the A_600_ of the culture had reached to approximately 1.0, L-arabinose was added (final concentration, 0.2%) and the bacteria were grown for additional 1 hour. The cultures were harvested, divided into supernatant and pellet fractions after centrifugation, and analyzed for the presence of TGFα-PE38 or PE38 by Western blotting with a polyclonal antibody against *Pseudomonas* exotoxin A (Figure 1B). *Salmonellae* carrying *pSEC-TGFa-PE38* were analyzed in the same manner to examine release of TGFα-PE38 through the Psp secretion signal peptide. In harvested whole cell cultures, the antibody detected TGFα-PE38 or PE38 protein only after addition of L-arabinose, demonstrating tight control by the *araBAD* promoter. Examination of the supernatant and pellet fractions after induction of the phage lysis system revealed that most of the target protein was present in the supernatant, demonstrating that the phage lysis system is effective. Note that SEC-TGFα-PE38 was detected in two forms, with and without a SEC signal peptide, in whole cell culture samples. However, only mature product lacking the signal peptide was observed in the supernatant fraction, while both forms were detected in the pellet fraction. The Psp signal peptide (MW 3.44 KDa) is cleaved upon translocation of the cargo protein out of the bacterial cell membrane. Taken together, these results show that engineered *Salmonellae* express and release TGFα-PE38 effectively in the presence of L-arabinose.

### Effect of TGFα-PE38 expressed by *Salmonella typhimurium* on cultured cancer cells

**Figure 2:**
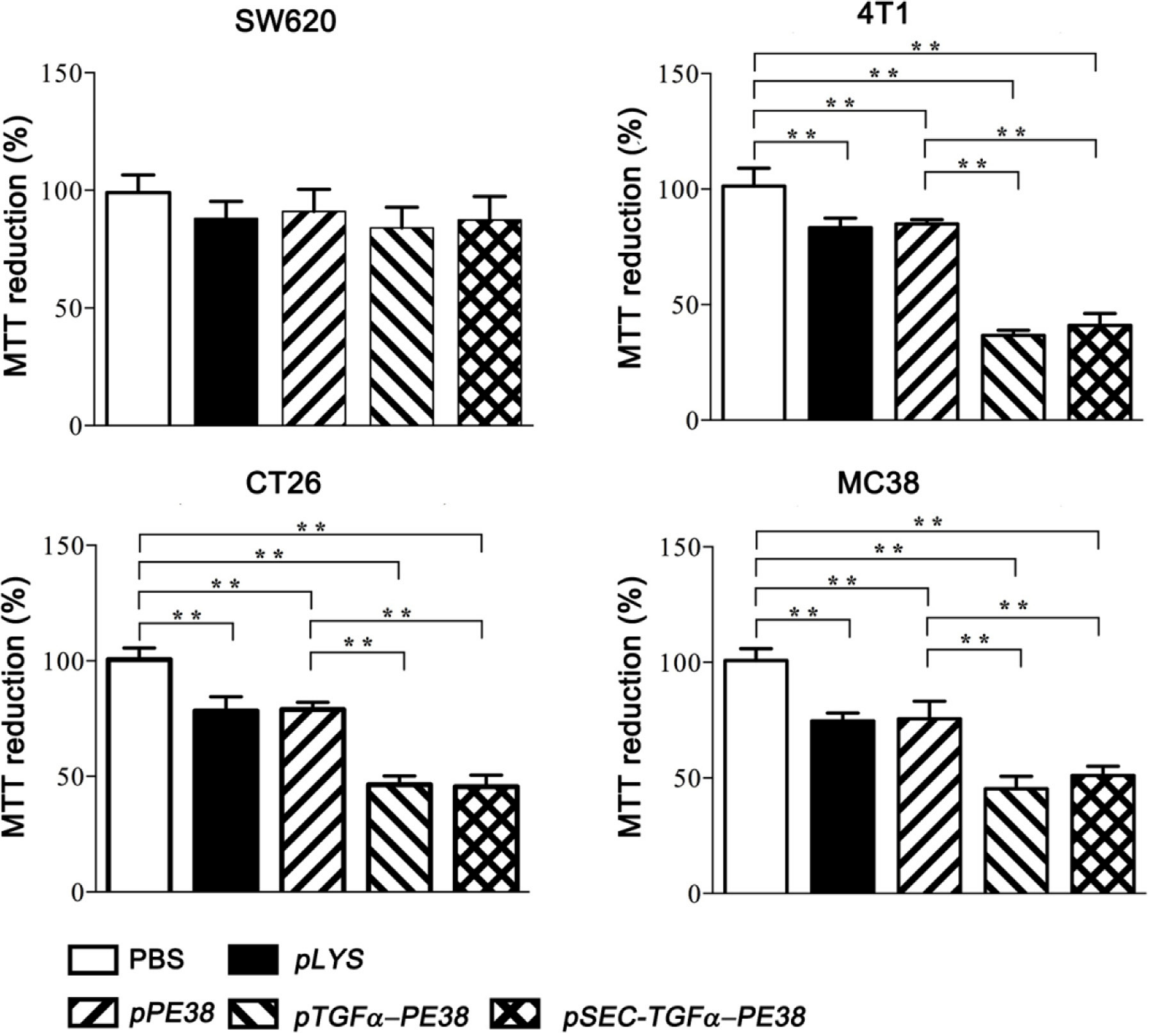
Fate of cancer cells treated for 24 hours with supernatant from a *Salmonella* culture containing TGFα-PE38 (released by the phage lysis system or by the secretory signal peptide) or PE38 Bacterial culture supernatant from *Salmonella* carrying the vector plasmid expressing the phage lysis system (*pLYS*) was also included in addition to PBS control. Cell death was assessed in a MTT assay. The degree of cell death was expressed relative to PBS-treated control (100%). Data are expressed as the mean ± SD (*n* = 4), and asterisks (*) indicate a significant difference compared with untreated controls (*P < 0.05).

TGFα-PE38 should be effective against EGFR-expressing cancer cells [41]. The effect was examined using two mouse colon cancer cell lines, CT26 and MC38, a mouse breast cancer cell line, 4T1, expressing considerable levels of EGFR [42, 43], and human colon cancer cells, SW620, expressing little EGFR [44]. Subsequently, TGFα-PE38/PE38 expressed and released from *Salmonella* into the culture medium was filtered through 0.45 μm pore filter, concentrated to 100 μg/ml, and tested on the above cultured tumor cells. After 24 hours of treatment, the cytotoxic effect of TGFα-PE38 was examined in an MTT assay (Figure 2). TGFα-PE38 in the filtrate was highly, although variably, cytotoxic to all tumor cell lines tested, with the exception of SW620 cells. Also, the effect was about the same irrespective of the source (phage lysis system or the signal peptide). PE38 lacking the cancer cell-binding moiety showed little cytotoxic effect. As a control, filtered media from induced cultures of the same *Salmonella* carrying the parental plasmid, *pLYS* were tested and found to be marginally toxic presumably owing to a contamination of bacterial endotoxin released upon bacterial lysis. Thus, we concluded that the cytotoxic effect conferred by TGFα-PE38 was mainly resulted from the cooperative effect of the EGFR-binding moiety and the toxin moiety of TGFα-PE38.

### Effect of TGFα-PE38 expressed by *Salmonellae* in mouse tumor models

**Figure 3:**
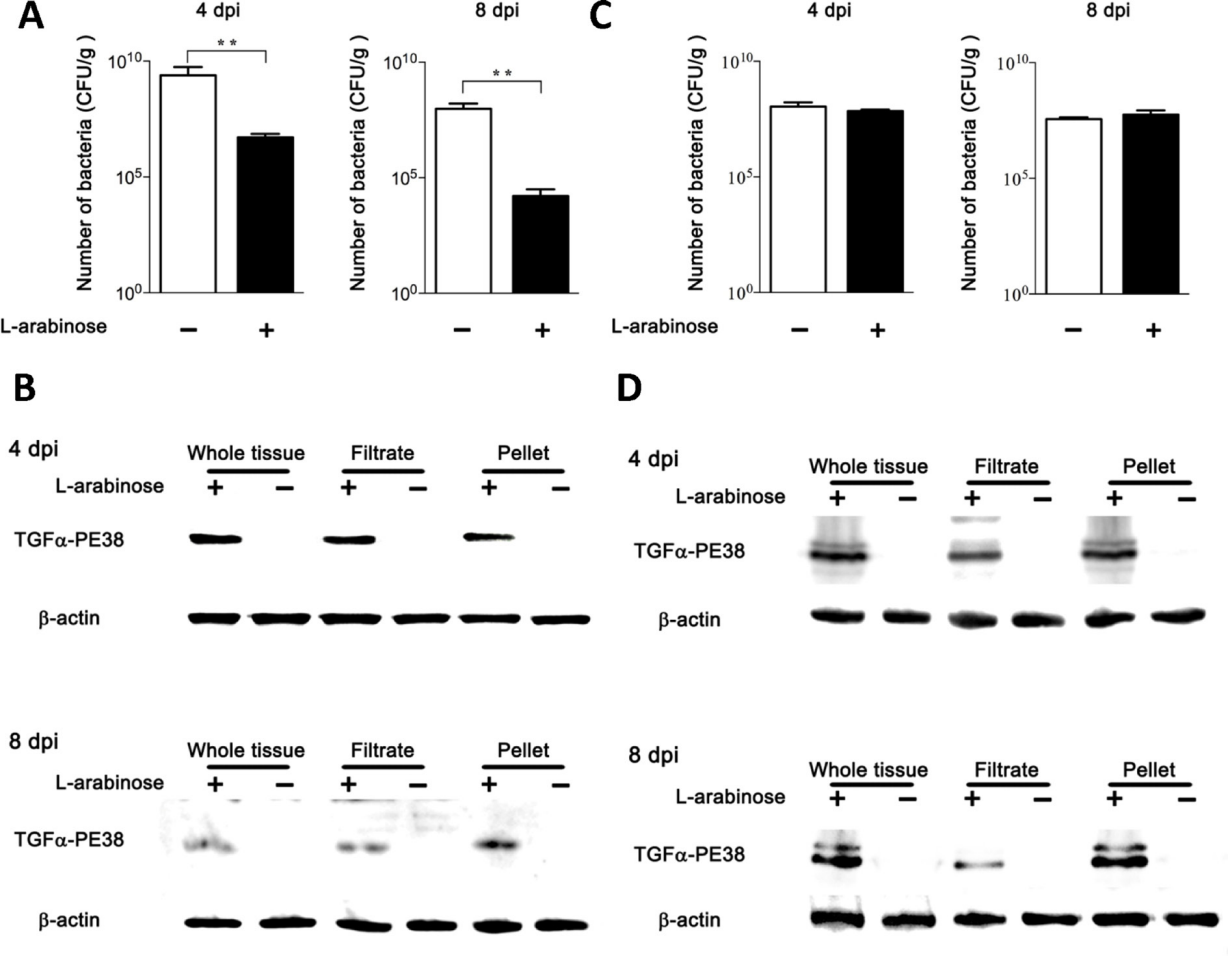
Expression and release of TGFα-PE38 from intra-tumoral *Salmonellae* (**A** and **B**) *Salmonellae* carrying *pTGFα-PE38* was injected into BALB/c mice implanted with CT26 cells via the tail vein. L-arabinose was administered into the peritoneal cavity at 3 and 7 days post-injection (*dpi*), and the number of *Salmonella* was determined at 4 and 8 *dpi*. Filled bars indicate L-arabinose treatment; open bars indicate no L-arabinose treatment (A). (B) Expression and release of TGFα-PE38 from intra-tumoral *Salmonellae* was determined 1 day after induction by L-arabinose administration(4 and 8 *dpi*). Whole tumor tissues were excised and separated into filtrate and pellet fractions. TGFα-PE38 was determined by Western blotting with a specific antibody. Panels (**C**) and (**D**) show result of treatment with *Salmonellae* carrying *pSEC-TGFα-PE38*. Total number of *Salmonellae* (C) and the expression and release of *SEC-TGFα-PE38* (D) were determined at indicated days.

The anti-tumor effects of TGFα-PE38 expressed and released from intra-tumoral *Salmonella* were determined in BALB/c mice bearing CT26 colon cancer cell xenografts. When the tumors reached to approximately 150 mm^3^ [20], ΔppGpp *Salmonella* (5 × 10^7^ Colony Forming Unit, CFU) carrying either *pTGFa-PE38* or *pSEC-TGFα-PE38* were injected through the tail vein. Three days post-injection (*dpi*), when the bacterial number in the tumor is highest [46], L-arabinose (120 mg) was administered into the peritoneal cavity. After 24 hours (i.e., 4 *dpi*), the mice were sacrificed, tumor tissues were excised, bacterial numbers were counted, and TGFα-PE38 expression was examined by Western blotting with a specific antibody. A second round of the induction was carried out by administering L-arabinose at 7 *dpi* and examining tumor tissues at 8 *dpi* (Figure 3A). The number of *Salmonella* carrying *pTGFa-PE38*, and thus equipped with the phage lysis system, in L-arabinose-treated tumors was ~ 10^6^ CFU/g tissue (CFU g^−1^) at 4 *dpi*, while that in untreated tumors was~10^9^ CFU g^−1^. The number of intra-tumoral *Salmonella* at 8 *dpi* (induced once at 3 and again at 7 *dpi*) was ~10^4^ CFU g^−1^, while that induced once (at 3 *dpi*) was ~10^6^ CFU g^-1^. These results suggested that the phage lysis system induced by L-arabinose resulted in > 99.9% bacterial lysis each time, consistent with our previous observations [34]. We also examined the expression and release of TGFα-PE38 by intra-tumoral *Salmonellae* (Figure 3B). Tumor tissues were homogenized and passed through a 0.45 μm pore filter to remove unlysed bacteria, and the filtrate and remaining cell pellet were separated and analyzed for TGFα-PE38 by Western blotting. TGFα-PE38 was detected only in L-arabinose-treated mice (Figure 3B, whole cells). A significant level of TGFα-PE38 was detected in the filtrate from tissue samples, indicating that this protein was released from intra-tumoral *Salmonellae*. The level of TGFα-PE38 after the second round of induction was considerably less than that after the first round, reflecting the reduction in bacterial numbers. The same experiment was carried out with *Salmonella* harboring *pSEC-TGFα-PE38*. In this case, the number of bacteria after two rounds of induction was hardly reduced (Figure 3C). When we analyzed expression of TGFα-PE38, we detected similar levels after the first and second inductions. It was also noted that released TGFα-PE38 lacking the signal peptide prevailed in the filtrate, while both forms of TGFα-PE38 were detected in the pellet. Thus, the Psp secretion signal works in the mouse model as well as *in vitro*. These results confirmed that *Salmonellae* carrying either *pTGFα-PE38* or *pSEC-TGFa-PE38* targeted grafted tumor tissue, where they expressed and released TGFα-PE38 specifically upon the administration of L-arabinose.

**Figure 4:**
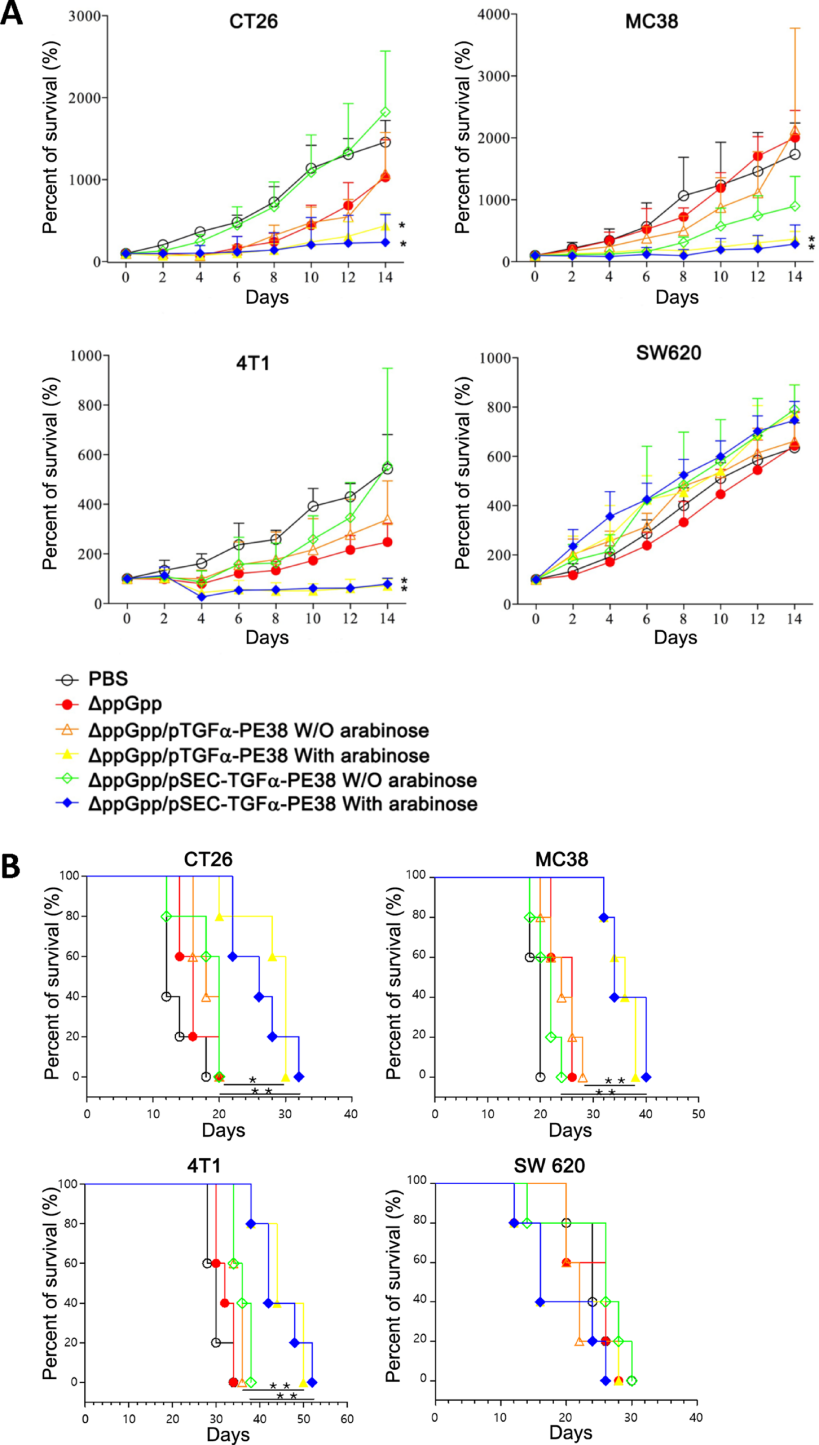
(**A**) Effects of *Salmonella* expressing TGFα-PE38 on mice implanted with different cancer cell lines (*n* = 5/group). BALB/c mice grafted with CT26 and 4T1 tumors, C57BL/6 mice with MC38 tumors, and nude mice with SW620, were analyzed. Percent changes in tumor sizes measured at indicated day relative to that at day 0 (100%) were plotted. (**B**) Kaplan-Meier survival curves for the tumor-bearing mice receiving the treatments described above (*n* = 5/group). Days in x-axis are after *Salmonella* injection. *P < 0.05; **P < 0.005.

Finally, we examined the anti-tumor activity of the engineered *Salmonella in vivo*. BALB/c mice were grafted with CT26 and 4T1 tumors, C57BL/6 mice with MC38 tumors, and nude mice with SW620. Tumor-bearing mice were then intravenous-injected with PBS, or 5 × 10^7^ CFU *Salmonella* alone or *Salmonella* carrying *pTGFα-PE38* with or without L-arabinose administration or with *Salmonella* carrying *SEC-TGFα-PE38* with or without L-arabinose administration. Tumor growth was then measured every 2 days (Figure 4A). Representative gross morphological changes of tumors are shown in Supplementary Figure 1. Treatment of tumor-bearing mice with *Salmonellae* carrying *pTGFα-PE38* or *pSEC-TGF*α*-PE38*, followed by induction with L-arabinose (at 3.5 *dpi* and at 7.5 *dpi*), led to significant retardation in the growth of all the tumor tissues except that of SW620: no discernable change was observed. The other treatments showed various degrees of anti-tumor activity depending on the tumor cell type except SW620, evidently better than the PBS control or *Salmonella* alone. We then measured the survival of those tumor-bearing mice treated with the same set of *Salmonella* (Figure 4B). The results showed that survival was dependent on retardation of tumor growth by *Salmonellae* carrying or expressing TGFα-PE38. Taken together, the data show that TGFα-PE38 released from intra-tumoral *Salmonellae* by the phage lysis system or by the secretion signal effectively retards tumor growth and improves survival.

## DISCUSSION

There are some elements that prevent immunotoxins, particularly TGFα-PE38, from treating EGFR-expressing tumors successfully. Among these, immunogenicity associated with PE38 derived from *Pseudomonas* has been virtually eliminated by introducing mutations [47]. However, in all preclinical trials the TGFα-PE38 protein was injected by continuous infusion, mainly due to its short half-life as well as hepatotoxicity [7, 8]. Here, however, we observed a significant effect against EGFR-expressing solid tumors when TGFα-PE38 was expressed and released by intra-tumoral *Salmonellae* (Figure 4). Previously, we compared the distribution of L-asparaginase, an anti-tumor protein used to treat acute lymphoblastic leukemia, between that expressed and secreted by tumor-targeted *Salmonellae* and that after intravenous injection [36]. In the latter case, a considerable amount of protein was detected in serum, but little in tumor tissue. By contrast, when mice were treated with *Salmonella* expressing L-asparaginase, the protein was detected exclusively in tumor tissue, with the amount increasing up to 48 hours after induction. It was suggested that tumor tissue provides an immuno-privileged environment, indeed a sanctuary, for intra-tumoral bacteria, which can then proliferate to yield up to 10^9^ CFU g^-1^ tissue [46, 48]. In addition, we speculate that the tumor tissue provides an environment in which protein drugs produced by intra-tumoral *Salmonellae* are protected from proteolytic degradation via host systems, thereby prolonging their efficacy. Thus, it should be possible to use other immunotoxins to treat various solid tumors using *Salmonellae* as a vehicle. It is certain that efficacy of immunotoxin would be greatly augmented when it is delivered by tumor-seeking bacteria.

Here, we used a signal sequence (Psp) to release TGFα-PE38 with the aim of achieving greater anti-tumor effects than those obtained with a phage lysis system: in this case, simultaneous induction of both TGFα-PE38 and the phage lysis gene by a single inducible *araBAD* promoter would reduce the anti-tumor effect because the number of intra-tumoral *Salmonellae* decreased after each round of induction. Indeed, on 16 *dpi*, after two rounds of induction, only a small numbers of *Salmonellae* were detectable in the tumor tissue (~20 CFU/g^−1^), while significant numbers were maintained in the absence of induction (~10^3^ CFU/g^−1^) (Supplementary Figure 2). Anti-tumor effect using TGFα-PE38 secreted through the signal sequence was only about the same as that observed after release via the phage lysis system. This suggests that it is the initial elicitation of a response by anti-tumor agents in the tumor mass, rather than prolonged exposure, that is critical for establishing an anti-tumor effect, particularly when using intra-tumoral *Salmonellae* as a drug carrier.

## MATERIALS AND METHODS

### Bacterial strains and culture conditions

ΔppGpp*S. typhimurium*, SHJ2037 (*relA::cat, spoT::kan*), has been previously described [32]. The strain carries an additional mutation in the *glmS* gene; therefore, it requires N-acetylglucosamine or D-glucosamine for growth if not complemented by a *glmS*-containing plasmid [36]. Bacterial cultures were grown in LB broth (Difco Laboratories) containing 1% NaCl with vigorous aeration at 37°C, unless otherwise indicated. For solid support medium, granulated agar (Difco Laboratories) was includedat 1.5%. Ampicillin (and other antibiotics) was purchased from Sigma Chemicals and added at the following concentration where necessary: 50 μg ml^−1^.

### Plasmids

All plasmids used in this study carried a *glmS* gene as the selective determinant of a balanced-lethal host vector system [36]. In addition, a *Salmonella* lysis system was also introduced into the plasmid, which comprised a gene from the *Salmonellae* bacteriophage, iEPS5 phage [34], except in the *pSEC-TGF*α*-PE38* plasmid. The DNA fragment of TGFα-PE38 or PE38 was PCR-amplified from plasmid pBR898, a generous gift from Dr. I. Pastan (NIH), as a template; this plasmid contains TGFα-PE38 under control of the T7 promoter [49]. Each fragment was cloned into the MCS of *pLYS* using the EcoR1 and Xba1 sites, to yield *pTGF*α*-PE38* or *pPE38*, respectively. The *Salmonella* SEC sequence was amplified from *Paenibacillus* sp. (BCCRC 17757) and then cloned into the plasmid containing no *Salmonella* lysis system but with a *glmS* gene on a pBAD24 backbone (*pSEC-TGF*α*-PE38*).

### Preparation of anti-tumor proteins expressed by *Salmonellae*

Bacterial cells were cultured overnight in LB broth and diluted 1:50 in fresh LB broth prior to incubation at 37°C. When the A_600_ reached 0.7, L-arabinose was added at a final concentration of 0.2%. Bacterial cultures were harvested by centrifugation at 3,000 × g for 15 min. The supernatant fraction was filtered through a 0.45 μm filter (SLHV033RS; Merck-Millipore) to remove bacterial cells and then concentrated using Centricon devices (Amicon Ultra 10 K; Millipore).

### Cell killing assays

Cell killing assays were performed using the tumor cell lines, CT26, 4T1, MC38 and SW620. Each cell line was seeded into 24-well plates at a density of 1 × 10^5^ cells per well. After 24 hours, the tumor cells were washed with PBS and placed in serum-free medium containing 500 μg of bacterial culture filtrate and induced to express and release TGFα-PE38 by addition of L-arabinose. After 24 hours, the number of surviving tumor cells was measured in a 3-(4,5-dimethylthiazol- 2-yl)-2,5-diphenyltetrazolium bromide (MTT) assay, as previously described [41].

### Western blot analysis

Total proteins were separated by electrophoresis on 10% SDS-PAGE gels and then transferred to a nitrocellulose membrane (Bio-Rad). After transfer, the membrane was blocked with 5% skim milk and probed with a rabbit anti-EGFR antibody (ab2430; Abcam), rabbit anti-*Pseudomonas* exotoxin A antibody (P2318; Sigma-Aldrich), or mouse anti β-actin antibody (sc-47778; Santa Cruz Technology) overnight at 4°C. After the primary antibodies were washed off with TBS (140 mM NaCl, 10 mM Tris-HCl, pH 8.0) containing 0.1% Tween 20, the membrane was incubated at room temperature for 1 hour with goat anti-rabbit IgG (ab6721; Abcam) or goat anti-mouse IgG (sc-2005; Santa Cruz Technology) conjugated to horseradish peroxidase. Bound proteins were visualized using an ECL kit (Amersham Biosciences).

### Culture of tumor cell lines

CT26 mouse colon carcinoma and 4T-1 mouse breast carcinoma cell lines were grown in high-glucose Dulbecco's Modified Eagle Medium containing 10% fetal bovine serum and 1% penicillin-streptomycin. MC38 cells were grown in Minimum Essential Medium, and SW620 human coloncarcinoma cells were grown in RPMI medium.

### Mouse experiments

Male BALB/c. C57BL/6 mice, and nude mice (5–6weeks old; 16–18g) were purchased from the Samtako Company, Korea. All animal care, experiments, and euthanasia were performed in accordance with protocols approved by the Chonnam National University Animal Research Committee. Tumor-bearing mice were generated by subcutaneously implanting cultured tumor cells (1 × 10^6^) suspended in 50 μl of PBS into the right thigh. After about 10–15 days, when tumors reached approximately 100–150 mm^3^, mice were injected (into tail vein) with *S. typhimurium* carrying various plasmids. Anesthesia prior to imaging was performed using isoflurane (2%). Anesthesia prior to surgery was performed using a mixture of ketamine (200 mg kg^−1^) and xylazine (10 mg kg^−1^).

### Counting bacterial cells in tumor tissue

Each solid tumor was excised from implanted mice and homogenized in 5 ml of PBS containing 0.05% Triton X-100. The homogenized tissues were diluted in PBS and plated on LB agar plates containing appropriate antibiotics. On the following day, bacterial numbers were counted on plates incubated at 37°C. The number of infecting bacteria was enumerated as follows: the number of bacteria × dilution factor/g of solid tumor tissue.

### Statistical analysis

Two-tailed Student's *t* tests were used to determine the significance of differences in primary tumor growth between the control and treated groups. For the MTT assay, statistical significance was determined using the Mann-Whitney *U* test. Survival analysis was performed using Kaplan-Meier curves and the log-rank test. *P* < 0.05 was considered significant for all analyses. All data are expressed as the mean ± SD.

## SUPPLEMENTARY MATERIALS FIGURES


