# Supplementary material for: Anti-tumor activity of an immunotoxin (TGFα-PE38) delivered by attenuated Salmonella typhimurium
Source: Oncotarget. 2017 Apr 18;8(23):37550–60. doi: 10.18632/oncotarget.17197 (PMC5514929; doi:10.18632/oncotarget.17197)
Supplement: Supplementary file 1 [file oncotarget-08-37550-s001.pdf]

Anti-tumor activity of an immunotoxin (TGFα-PE38) delivered by attenuated *Salmonella typhimurium*

Supplementary Materials

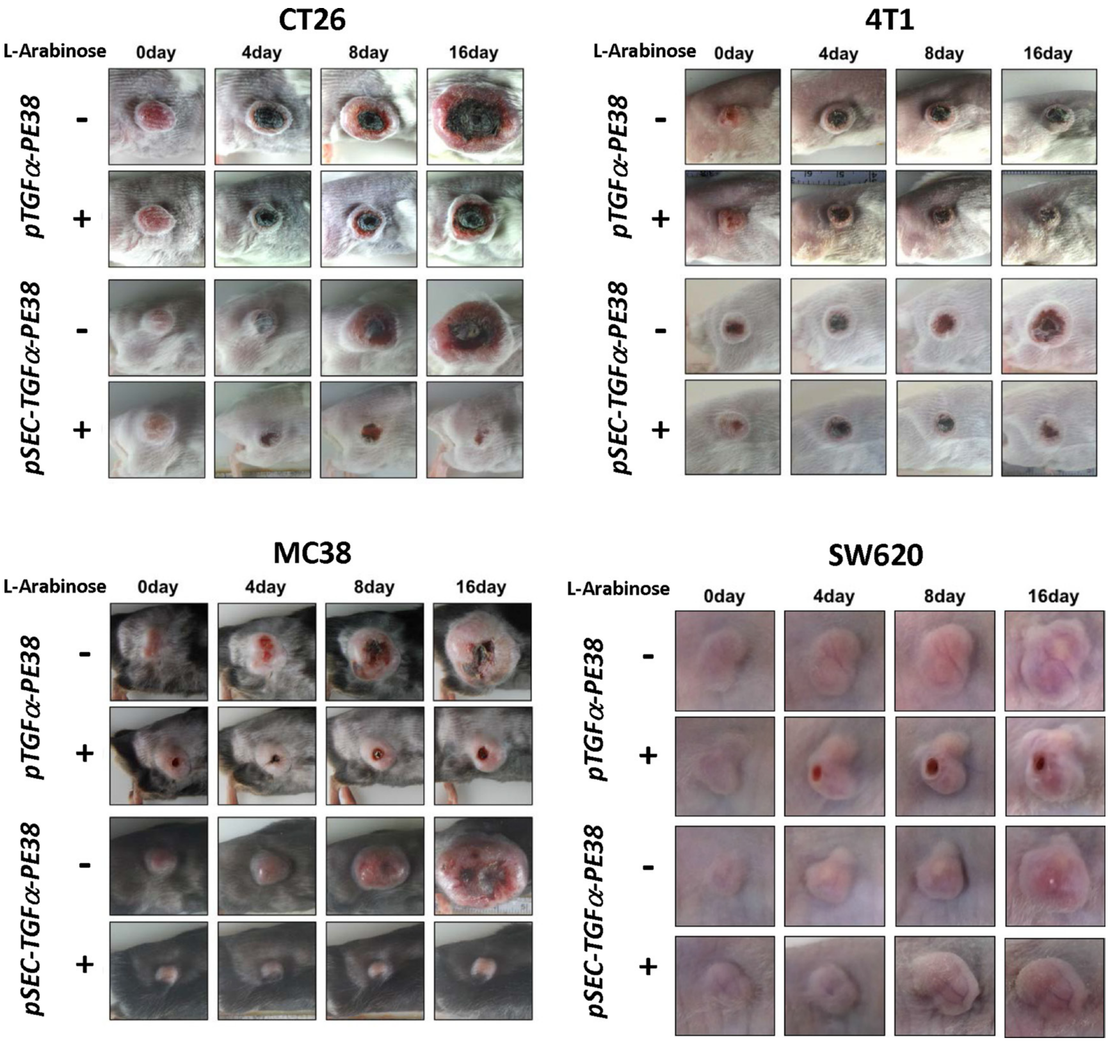

Supplementary Figure 1: Representative gross morphological changes of tumors shown in Figure 4.

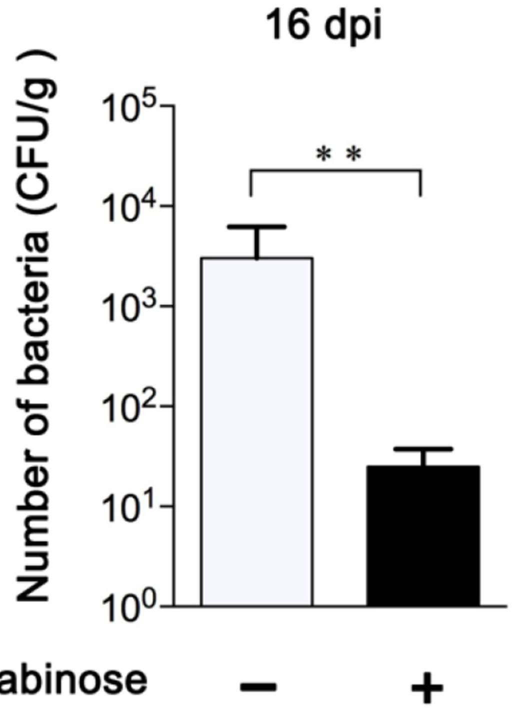

Supplementary Figure 2: Number of intra-tumoral *Salmonella* on 16 *dpi* after two rounds of inductions. CT26-bearing BALb/c mice were used.
